# Simplified Triceps Surae Muscle Volume Assessment in Older Adults

**DOI:** 10.3389/fphys.2019.01299

**Published:** 2019-10-10

**Authors:** Kiros Karamanidis, Gaspar Epro, Matthias König, Falk Mersmann, Adamantios Arampatzis

**Affiliations:** ^1^School of Applied Sciences, Sport and Exercise Science Research Centre, London South Bank University, London, United Kingdom; ^2^Department of Training and Movement Sciences, Humboldt-Universität zu Berlin, Berlin, Germany; ^3^Berlin School of Movement Science, Humboldt-Universität zu Berlin, Berlin, Germany

**Keywords:** magnetic resonance imaging, aged, muscle reconstruction, muscle volume, shape factor

## Abstract

Triceps surae (TS) muscle volume can be estimated in young adults by only considering the maximal anatomical cross-sectional area (ACSA_max_) and the length of the muscle due to the presence of a constant muscle-specific shape factor. This study aimed to investigate if this simplified muscle volume assessment is also applicable in older adults or if muscle-specific shape changes with aging. MRI sequences were taken from the dominant leg of 21 older female adults. The boundaries of all three TS muscles (SOL, soleus; GM, gastrocnemius medialis; GL, gastrocnemius lateralis) were manually outlined in transverse image sequences, and muscle volume for each muscle was calculated as the integral of the obtained cross-sectional areas of the contours along the whole length of the muscle (measured volume) and, in addition, by using the average muscle-specific shape factors of each muscle obtained from the ratio of the measured volume and the product of ACSA_max_ and the muscle length (estimated volume). There were no differences in the measured and estimated muscle volumes (SOL: 357.7 ± 61.8 vs. 358.8 ± 65.3 cm^3^; GM: 179.5 ± 32.8 vs. 179.8 ± 33.3 cm^3^; GL: 90.2 ± 15.9 vs. 90.4 ± 14.8 cm^3^). However, when using the reported shape factors of younger adults instead, we found a significant (*p* < 0.05) overestimation of muscle volume for SOL and GM with average RMS differences of 6.1 and 7.6%, respectively. These results indicate that corrections of muscle-specific shape factors are needed when using the previously proposed simplified muscle volume assessment as aging may not only be accompanied with muscle atrophy but also changes in the shape of skeletal muscle.

## Introduction

Muscle volume reportedly undergoes tremendous changes with maturation (O’Brien et al., 2010), several pathologies (Zoabli et al., 2008; Ji et al., 2013), mechanical loading (Folland and Williams, 2007), immobilization (Oates et al., 2010), or aging (Morse et al., 2005a). This is of functional relevance as muscle volume is an important determinant of the muscle mechanical power (O’Brien et al., 2009) and hence physical performance (Chelly and Denis, 2001). Another relevant component of physical performance is the maximum force generating capacity of a muscle, which is mainly determined by the physiological cross-sectional area (PCSA; Haxton, 1944; Aagaard et al., 2001; Fukunaga et al., 2001). In pennate muscles, it is not possible to measure the cross-sectional area PCSA *in vivo*; however, the indirect calculation by dividing the muscle volume by fascicle length (Powell et al., 1984; Lieber and Fridén, 2000) is well accepted, yet also reliant on muscle volume assessment. Thus, muscle volume is a crucial measure for investigating the mechanisms behind the physical capacity in different populations and evaluating the effectiveness of different interventions to enhance muscle function related to changes in muscle morphology.

The assessment of muscle volume usually involves the segmentation of the muscle from magnetic resonance imaging (MRI) recordings, which is a time-consuming procedure and, therefore, often limited for the application in clinical or research settings. Regarding this issue, Albracht et al. (2008) introduced a simplified method for the assessment of human triceps surae (TS) muscle volume *in vivo*. The approach is based on the assumption that muscle volume can be calculated as the product of the muscle length and average anatomical cross-sectional area (ACSA) with the latter being a constant muscle shape-dependent fraction (i.e., shape factor) of the maximal ACSA (Albracht et al., 2008). Assuming the muscle-specific shape to be relatively constant across populations, TS muscle volume calculation only requires the determination of the relatively easy assessable individual maximum ACSA and muscle length. Indeed, in the study of Mersmann et al. (2014), TS shape factors of young untrained, endurance, and strength trained adults were found to be in a good agreement, despite large differences in corresponding muscle volumes. Hence, gastrocnemius medialis (GM), gastrocnemius lateralis (GL), and soleus (SOL) muscle volume of an independent group of recreationally active subjects were precisely estimated using the corresponding average shape factors from the mentioned three groups. However, in order to provide a prospect for the application of the reported time-saving assessment method in scientific and clinical settings, it is further necessary to determine whether the TS muscle shape features a similar consistency across different age populations. As there is evidence that aging leads to significant changes in muscle architecture (e.g., Narici et al., 2003) and affects the muscle volume distribution at the lower limbs (e.g., Thom et al., 2005), it seems reasonable to suggest that there might be differences in muscle shape between young and older adults. Moreover, there is proof for an inhomogeneous TS muscle atrophy along the length of the muscle due to immobilization (Akima et al., 2000; Miokovic et al., 2012), which would affect the ratio of average to maximum ACSA (i.e., shape factor). Accordingly, the generalizability of the reported TS shape factors of young adults’ muscles to muscles that underwent atrophy due to aging cannot be assumed *a priori* and needs to be verified.

Therefore, the aim of the present study was to investigate if the previously proposed simplified muscle volume assessment method (i.e., shape factor-based assessment) for the TS muscle group (i.e., SOL, GM, and GL) is also valid for older adults. As a second step, we aimed to cross-validate the shape factor reported by Albracht et al. (2008) with the TS morphology data determined from the current group of older adults to extend the examination of TS muscle shape consistency across different age groups. We hypothesized that the assessment of muscle volume using the maximum ACSA and muscle length in older adults and the shape factors provided by the literature from young adults (Albracht et al., 2008) would provide discrepancies in the assessment of muscle volume, and an age-specific correction of the shape factors is required.

## Materials and Methods

### Participants

The study was conducted with 21 healthy older female adults aged between 60 and 75 years [age: 65 ± 7 years; body mass: 63 ± 9 kg; body height: 165 ± 5 cm; mean; and standard deviation (SD)] who agreed to have their dominant limb scanned using MRI. Exclusion criteria were any musculoskeletal or neurological impairments of the lower limbs or pain during daily life within the last 2 years, which might influence the findings of the current study. The participating older adults were recreationally physically active and representative for their age group (mean outcome of the SF-36 general health questionnaire of 69.9%; average single leg stance time of 43.0 s out of maximal 45 s test duration; mean timed up and go test result of 7.1 s). The study was approved by the responsible ethics committees (German Sport University Cologne), and all participants provided their written informed consent after being informed about the procedures and possible risks.

### Data Acquisition

**Figure 1:**
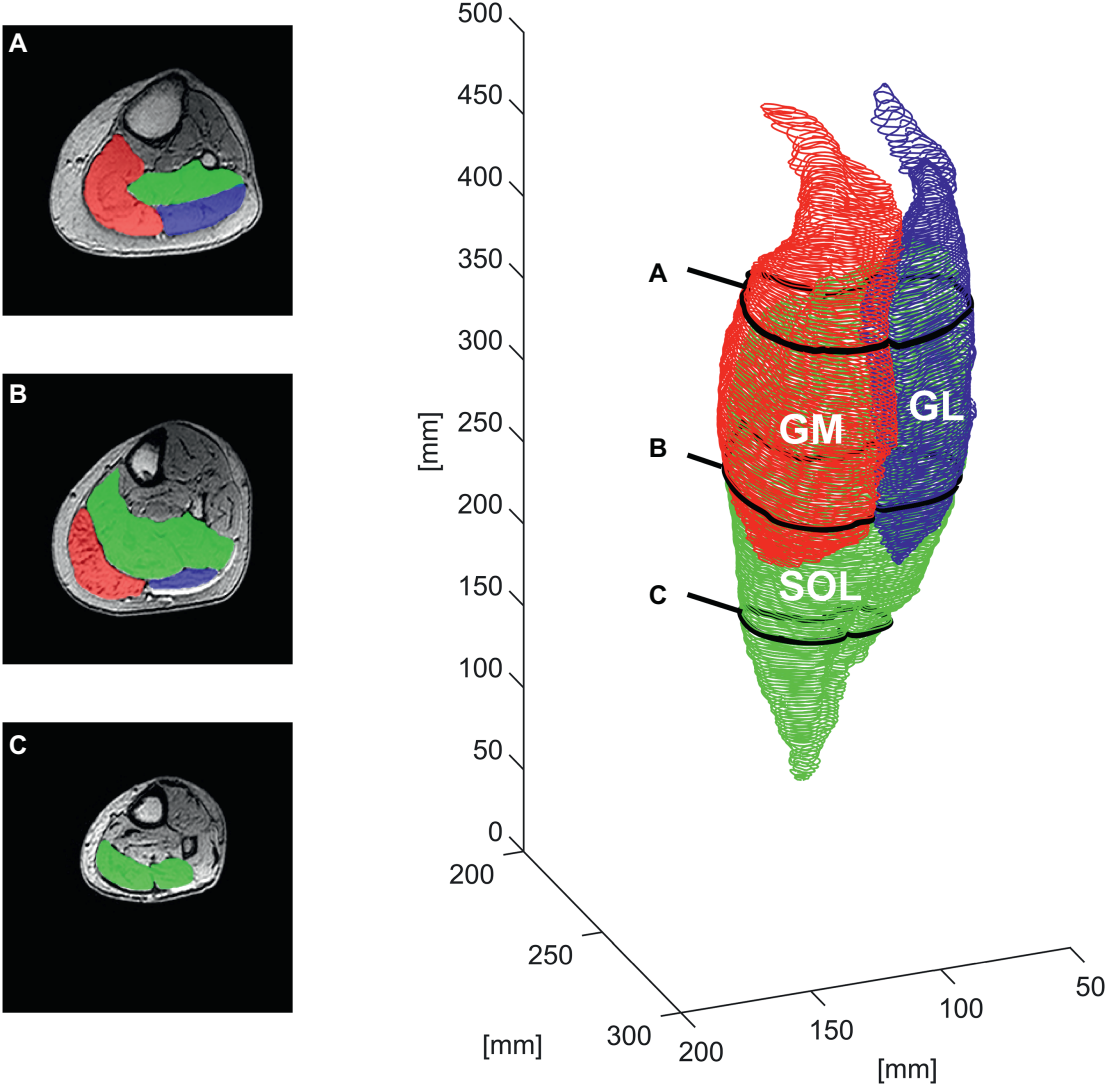
Manual segmentation of the contours of the gastrocnemius medialis (GM), gastrocnemius lateralis (GL), and soleus muscle (SOL) for one representative older adult in the transverse plane at ∼85% **(A)**, ∼60% **(B)**, and ∼30% **(C)** of the shank length (left) and the respective whole-muscle reconstruction (right).

Image sequences of the lower limb were acquired with a 3 Tesla MRI scanner in transverse and sagittal plane between the femur condyles and the calcaneal tuberosity in an unloaded supine position with the hip and knee fully extended and the ankle joint fixed at 20° plantar flexion (TS muscle-tendon unit close to slack position; De Monte et al., 2006). The sequences were acquired with a slice thickness of 1.0 mm, no inter-slice spacing. The sagittal sequences were recorded for the later analysis of the anatomical landmarks (origin and insertion) of all three TS muscles (i.e., SOL, GM, and GL; see Figure 1).

To measure the volume of the TS, the boundaries of individual muscles (SOL, GM, and GL) were manually outlined in every second transverse image at every 2 mm along the whole muscle length between the two marginal slices using a custom routine of the image processing program ImageJ (ImageJ 1.48v; National Institutes of Health, USA). In order to prevent an overestimation of TS, muscle mass, subcutaneous fat, vessels, tendon, and aponeuroses were excluded in the segmentation process (Fukunaga et al., 1992). The acquired coordinates and contours were exported as 3D coordinates and further processed using custom routines in MATLAB 2018a (The MathWorks, Natick, MA, USA; see Figure 1). The resulting contours were used to calculate the muscle volume (V_measured_) as the integral of the obtained ACSA of the contours along the whole length of the muscle (L_muscle_), which was determined as the distance between the two marginal slices contributing to muscle reconstruction along the longitudinal axis of the coordinate system (distance between the obtained transverse images).

### Investigation of Muscle-Specific Shape and Muscle Volume Estimation

Based on the proposed theoretical consideration of Albracht et al. (2008) that V_measured_ is the product of its mean ACSA and L_muscle_, according to which the mean ACSA may be described as a fraction (*p*; i.e., shape factor) of the maximum ACSA (ACSA_max_), the individual TS shape factors were obtained for older adults from the assessed muscle reconstructions by dividing the measured muscle volume by the product of ACSA_max_ and the L_muscle_:

(1) $$p=\frac{V_{\mathrm{measured}}}{{\mathrm{ACSA}}_{\text{max}}\times L_{\mathrm{muscle}}}$$

In order to evaluate the applicability of the muscle volume assessment (V_estimated_) based on the newly calculated muscle shape, the V_measured_ obtained by whole-muscle segmentation was compared to those predicted based on Eq. (2) using the measured ACSA_max_ and the L_muscle_, and the average shape factor obtained from the whole group of analyzed older adults.

(2) $$V_{\mathrm{estimated}}=p\times {\mathrm{ACSA}}_{\text{max}}\times L_{\mathrm{muscle}}$$

In addition, we aimed to examine whether the previously reported muscle shape factors for younger subjects (Albracht et al., 2008) are valid for muscle volume assessment in the elderly. We therefore cross-validated the previously reported shape factor and compared the measured volumes to volumes estimated from Eq. (2) using measured ACSA_max_ and L_muscle_ values from the present data set of older adults and average shape factors (for each investigated muscle) calculated from the group of healthy younger adults reported by Albracht et al. (2008); *n* = 13; age: 29 ± 6 years; body mass: 76 ± 6 kg; body height: 180 ± 4 cm; average shape factors: 0.496, 0.592, and 0.569 for SOL, GM, and GL, respectively.

### Statistics

A one-way analysis of variance (ANOVA) with investigated muscle (i.e., SOL, GM, and GL) as factor was used to detect potential differences in the analyzed muscle morphological characteristics [muscle specific shape factor (*p*), V_measured_, ASCA_max_, position of the maximum ACSA relative to the shank length, and L_muscle_] between the three components of the TS in our group of older adults. A Bonferroni *post hoc* test was applied to identify potential differences between the three compartments of the TS regarding the muscle-specific shape factor *p*, V_measured,_ ASCA_max_, the position of the maximum ACSA relative to the shank length, and L_muscle_. Inter-subject variability of shape factors and the position of the maximal ACSA relative to the shank were calculated using the coefficient of variance.

For the validation of the muscle volume assessment based on the muscle shape, the estimated muscle volume (i.e., SOL, GM, and GL) using the shape factors obtained from the current pool of older adults and the one measured from the whole muscle MRI analysis was compared by means of a paired sample *t* test after checking for normal distribution using the Kolmogorov-Smirnov test. For accuracy evaluation, the root mean squares (RMSs) of the differences between estimated and measured volume as well as the coefficient of determination (*R*^2^) were calculated. To further test the validity of the shape factor-based assessment and whether the TS muscle shape factors feature a similar consistency across age populations, we cross-validated the muscle shape factors of younger subjects reported by Albracht et al. (2008) with the determined TS volume using the same statistical procedure as implemented using the muscle shape factors of older adults. The level of statistical significance was set at *α* = 0.05, and all results in the text, tables, and figures are presented as mean and SD. All statistical analyses were performed using Statistica software (release 10.0; Statsoft, Tulsa, OK, USA).

## Results

### Triceps Surae Muscle Morphology

**Figure 2:**
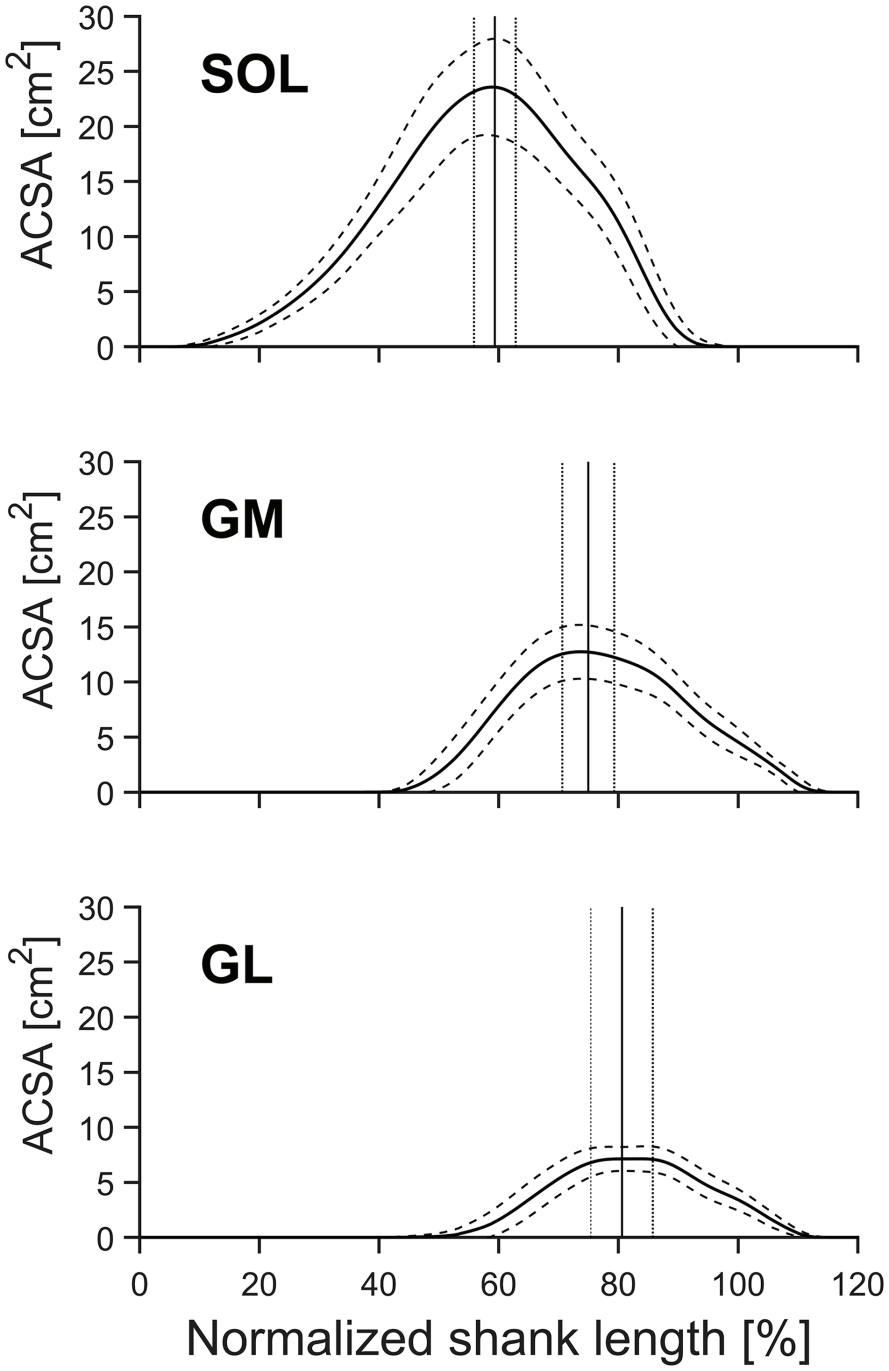
Mean anatomical cross-sectional area (ACSA) and standard deviation (SD) of the gastrocnemius medialis (GM), gastrocnemius lateralis (GL), and soleus muscle (SOL) of older adults (*n* = 21) as a function of relative shank length [from distal (0%) to proximal (100%)]. The vertical lines indicate the mean position ± SD (dotted lines) of the maximum ACSA of the pooled data. Solid lines indicate the means, and dashed lines indicate the SD.

A significant (*p* < 0.01) muscle effect was found for all analyzed morphological parameters within the TS (muscle specific shape factor *p*, V_measured_, ACSA_max_, position of the maximum ACSA relative to the shank, and L_muscle_) with the SOL showing the largest muscle length, ACSA_max_, and volume, followed by the GM and GL (Table 1, Figure 2). Muscle-specific shape factor displayed significantly (*p* < 0.05) smaller values for SOL (0.484 ± 0.027) in comparison to GM (0.556 ± 0.028) and GL (0.568 ± 0.049), whereas no differences between the two gastrocnemii muscles were detected. The inter-subject variability of the shape factors, described by the coefficient of variation, showed low values for all analyzed muscles with 5.6, 5.0, and 8.6% for SOL, GM, and GL, respectively. Furthermore, the maximum ACSA was located at 59.3 ± 3.5, 75.0 ± 4.3, and 80.6 ± 5.1% of the shank length (measured from tuberositas calcanei to the tibial plateau) for the SOL, GM, and GL, respectively (significantly different locations between all muscles; see Figure 2), and the inter-subject variability (coefficient of variance) of the ACSA_max_ location ranged between 5.8 and 6.4%.

**Table 1:** Means ± standard deviations of the maximal anatomical cross-sectional area (ACSA_max_), measured muscle volume (V_measured_) and estimated muscle volume with shape factor from current older adults (V_estimated OLD_) and young adults (V_estimated YOUNG_) of the soleus (SOL), gastrocnemius medialis (GM) and gastrocnemius lateralis muscles (GL).

Muscle	L_muscle_ (cm)	ACSA_max_ (cm^2^)	V_measured_ (cm^3^)	V_estimated OLD_ (cm^3^)	V_estimated YOUNG_ (cm^3^)
SOL	30.8 ± 0.7	24.0 ± 4.4	357.7 ± 61.8	358.8 ± 65.3	367.7 ± 66.9^a^
GM	24.7 ± 1.4^*^	13.1 ± 2.3^*^	179.5 ± 32.8^*^	179.8 ± 33.3	191.4 ± 35.5^a^
GL	21.2 ± 2.1^*^^,^^#^	7.6 ± 1.2^*^^,^^#^	90.2 ± 15.9^*^^,^^#^	90.4 ± 14.8	90.5 ± 14.9

* *Statistically significant (p < 0.05) differences to SOL*.

# *Statistically significant (p < 0.05) differences to GM*.

a *Statistically significant (p < 0.05) differences to V_measured_*.

### Triceps Surae Muscle Volume Assessment

**Figure 3:**
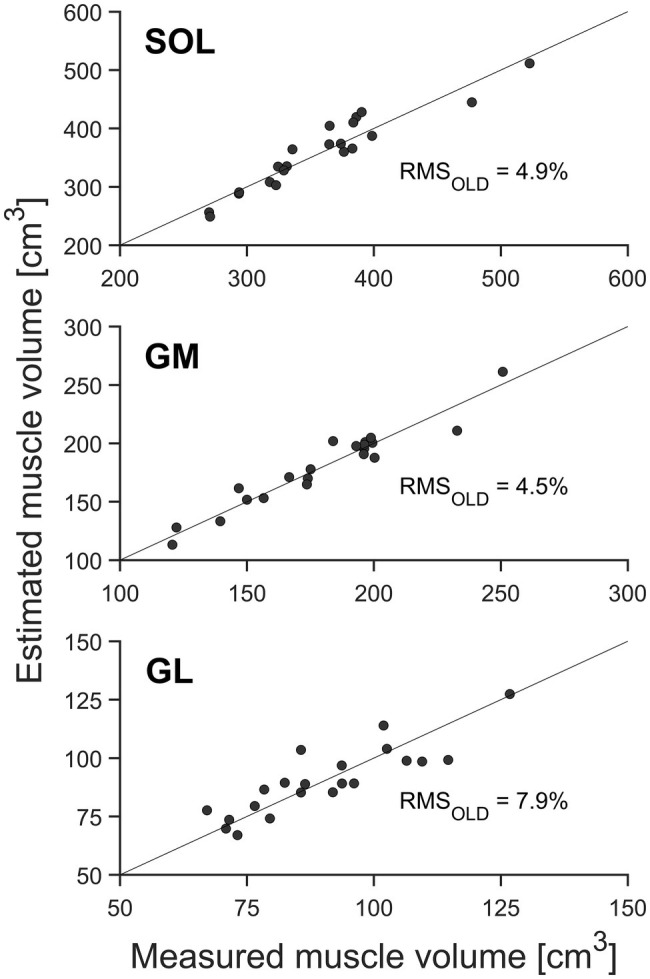
Muscle volume of the gastrocnemius medialis (GM), gastrocnemius lateralis (GL), and soleus muscle (SOL; *n* = 21) by means of whole-muscle segmentation (abscissa) in relation to estimated muscle volume using the muscle-specific shape factors of older adults (ordinate). The solid diagonal line represents the identity line. The relative root mean square (RMS) of the differences between methods is included in the figure.

There were no significant differences between the muscle volumes obtained from whole-muscle segmentation and the volumes predicted using the measured ACSA_max_, muscle length, and the average shape factors obtained from the current pool of older adults (Table 1, Figure 3). Furthermore, the coefficient of determination (*R*^2^) for the assessed muscle volumes using the newly calculated shape factors from older adults was quite high for all three muscles (0.90, 0.92, and 0.74, respectively, for SOL, GM, and GL). The relative RMS difference between the measured and estimated muscle volumes using the newly calculated shape factors from the current older adults was 4.9, 4.5, and 7.9% for SOL, GM, and GL, respectively (Figure 3).

**Figure 4:**
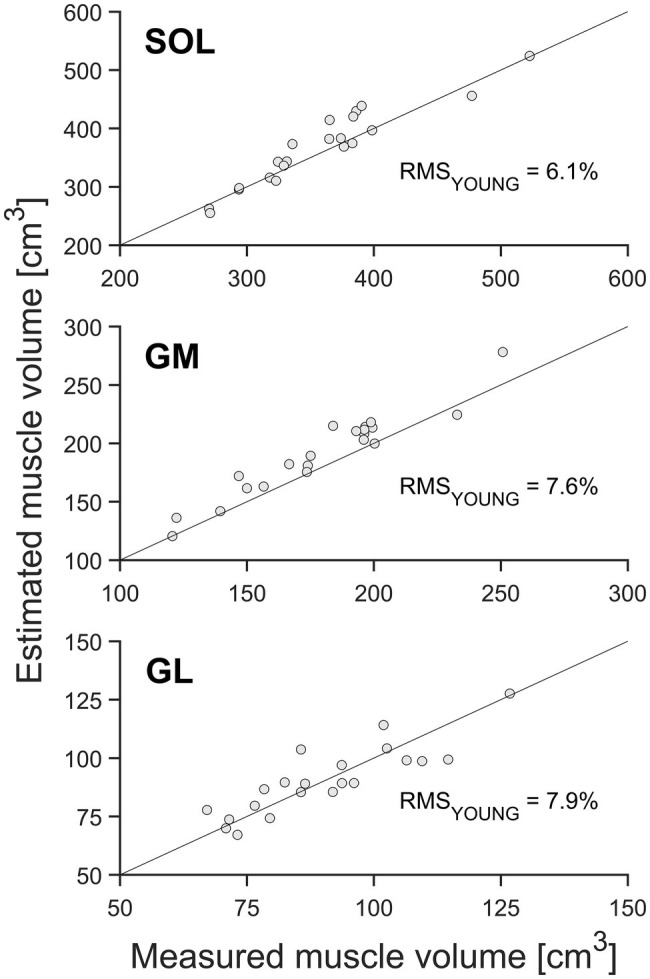
Muscle volume of the gastrocnemius medialis (GM), gastrocnemius lateralis (GL), and soleus muscle (SOL; *n* = 21) by means of whole-muscle segmentation (abscissa) in relation to estimated muscle volume using the muscle-specific shape factors of the young adults (ordinate). The solid diagonal line represents the identity line. The relative root mean square (RMS) of the differences between methods is included in the figure.

When estimating muscle volume by using the shape factors previously reported by Albracht et al. (2008) for younger adults (0.496, 0.592, and 0.569 for SOL, GM, and GL, respectively), a significant (*p* < 0.05) overestimation of the muscle volume was detected for SOL and GM compartments of the TS compared to the volumes measured from whole-muscle reconstruction (with relative RMS differences of 6.1% for SOL and 7.6% for GM; Table 1, Figures 3, 4). No significant differences between the measured muscle volume and either of the estimated muscle volumes were detected for the GL and relative RMS differences of 7.9% (Table 1, Figures 3, 4). Because the individual values of ACSA_max_ and L_muscle_ were the same for both estimation approaches, the coefficients of determination did not differ as well (Figures 3, 4).

## Discussion

Previous studies reported that it is possible to assess the individual muscle volume within the TS muscle group in younger adults by only using the maximal ACSA, the length of the muscle, and a muscle-specific shape factor (Albracht et al., 2008; Mersmann et al., 2014). However, whether this simplified method is also valid for muscles that underwent atrophy due to aging is not established. The current findings suggest that the previously proposed simplified approach for assessing muscle volume by using shape factors is applicable for scientific or clinical use in the older population. However, the results also indicate that using the muscle-specific shape factor from younger adults partly overestimates the muscle volume in the TS muscles in the elderly, providing evidence that aging may not only be accompanied with muscle atrophy but also by changes in muscle shape.

Albracht et al. (2008) reported that the size of the ratio between muscle volume and the product of maximal ACSA and length of the muscle (i.e., the shape factor *p*) is muscle specific and hence not consistent within the TS muscle in young adults. In accordance with these findings, the data of our older population revealed that the value of *p* was significantly different between the SOL (0.484 ± 0.027) and both gastrocnemii muscles (GM: 0.556 ± 0.028; GL: 0.568 ± 0.049), whereas no significant difference was detected between the GM and GL (indicating a similar ratio of average and maximum ACSA). However, despite these differences within the TS, we found a considerably low inter-subject variability for *p* (coefficient of variance between 5.0 and 8.6%), thus indicating that the shape of each muscle seems to be similar across the examined population of older adults. Due to the markedly low inter-subject variability of the shape factor *p*, the assessment of muscle volume from ACSA_max_ and muscle length was possible with a generally good agreement (coefficients of determination for the assessed muscle volume were quite high) and relative RMS differences between 4.5 and 7.9%. These error of prediction and coefficient of determination values within our group of older adults are in accordance with the values reported in younger adults (Albracht et al., 2008), showing that the previously proposed simplified muscle volume assessment for the TS muscle group is applicable for older adults.

It is important to note, however, that the relative RMS difference of 7.9% between measured and estimated GL muscle volume was clearly higher than the corresponding value for the SOL (4.9%) or GM (4.5%) muscle. Higher error of prediction for the GL in comparison with SOL or GM has also been previously reported in young adults (Albracht et al., 2008), indicating that the error of prediction is muscle but not age dependent. The absolute volume of the GL is clearly lowest (e.g., approximately a quarter of the size of the SOL), and we argue that the GL is therefore more vulnerable to occurring errors in the manual muscle ACSA segmentation leading to higher relative errors. Nevertheless, the previously reported values of relative volume changes following strength training or sarcopenia have been considerably larger for all TS muscles (Morse et al., 2005a,b). For example, aging has been found to be associated with decreases in TS muscle volume of 17–29% (Thom et al., 2005; Morse et al., 2005a). Further, Morse et al. (2005b) reported an average increase in 11, 15, and 19% for SOL, GM, and GL muscle volume, respectively, following a 12-month resistance training program in older adults. Based on the above results, we can conclude that a simplified assessment method using the muscle shape factor, the muscle length, and its maximum ACSA for muscle volume estimation is sensitive enough to detect exercise-related hypertrophic responses of the plantar flexors as well as muscle atrophy-related changes induced by aging.

In contrast to the above findings, when using the shape factors reported in the literature from younger adults (Albracht et al., 2008), we found a significant overestimation of the predicted muscle volume for SOL and GM compared to the values measured based on the whole-muscle reconstruction. Further, the prediction errors for the GM (7.6%) and SOL (6.1%) muscle volume were larger using shape factors from young adults compared to those using the average shape factor from the older adults (4.9 and 4.5%, respectively). Thus, the assessment of muscle volume using the maximum ACSA, muscle length in older adults, and the shape factors provided by the literature from young adults (Albracht et al., 2008) provides less accurate results (at least for SOL and GM) than using the newly obtained shape factor, indicating that muscle-specific shape factors may change with aging. Accordingly, the generalizability of the reported TS shape factors of young adults’ muscles to atrophied muscles of older adults could not be verified, and TS muscle shape consistency across different age groups is not given. This suggestion is further supported by the fact that the inter-subject variability (coefficient of variance) of SOL (5.6%) and GM (5.0%) muscle shape factors within our group of older adults was considerably small and is in accordance with the inter-subject variability values previously reported in young adults (Albracht et al., 2008). This means that the change in muscle shape suggested by our results seems to be a quite consistent age-related development.

Alterations in muscle shape might occur due to an inhomogeneous muscle atrophy along the length of the muscles, which has been previously reported for the TS muscle following several weeks of immobilization (Akima et al., 2000; Miokovic et al., 2012). For instance, Miokovic et al. (2012) demonstrated that atrophy of the GM and GL was greatest in their distal parts, which may lead to lower shape factors. Further, when considering all plantar flexor muscles as a whole, there was a tendency toward distal portions of the TS to be more affected during bed rest with no-exercise (Akima et al., 2000). Although it remains questionable whether such unloading paradigms appropriately represent the process of age-related atrophy (in terms of non-uniformity), these results together with the current findings suggest that aging may not only be accompanied with general loss of muscle mass but also with changes in the shape of skeletal muscle. Regarding this issue, various studies have shown that human muscles are divided into architectural subregions (Segal et al., 1991) and demonstrate region-specific differences in fiber type distribution (Johnson et al., 1973; Elder et al., 1982) as well as distinct innervation patterns (Yang et al., 1998; Buckland et al., 2009). Hence, during human movement, these different muscle subdivisions may have slightly different functions. As older than young adults show altered locomotion mechanics and ankle joint kinetics (Karamanidis and Arampatzis, 2007), it seems possible that specific regions of the GM and SOL muscle are recruited differently causing an inhomogeneous TS muscle atrophy in response to aging.

It is important to note that in the current investigation on a group of older adults, the position of the maximum ACSA was at 59.3 ± 3.5, 75.0 ± 4.3, and 80.6 ± 5.1% of the shank length for SOL, GM, and GL, respectively, and showed low inter-individual variability (range between 5.8 and 6.4%). Due to the unimodal distribution of the muscle ACSA, the ACSA_max_ can easily be identified with a few segmentations at the approximated positions (see Figure 2). Therefore, calculating the muscle volume using the proposed method and shape factors for older adults greatly reduces the required time for muscle volume assessment compared with full muscle segmentation.

**Figure 5:**
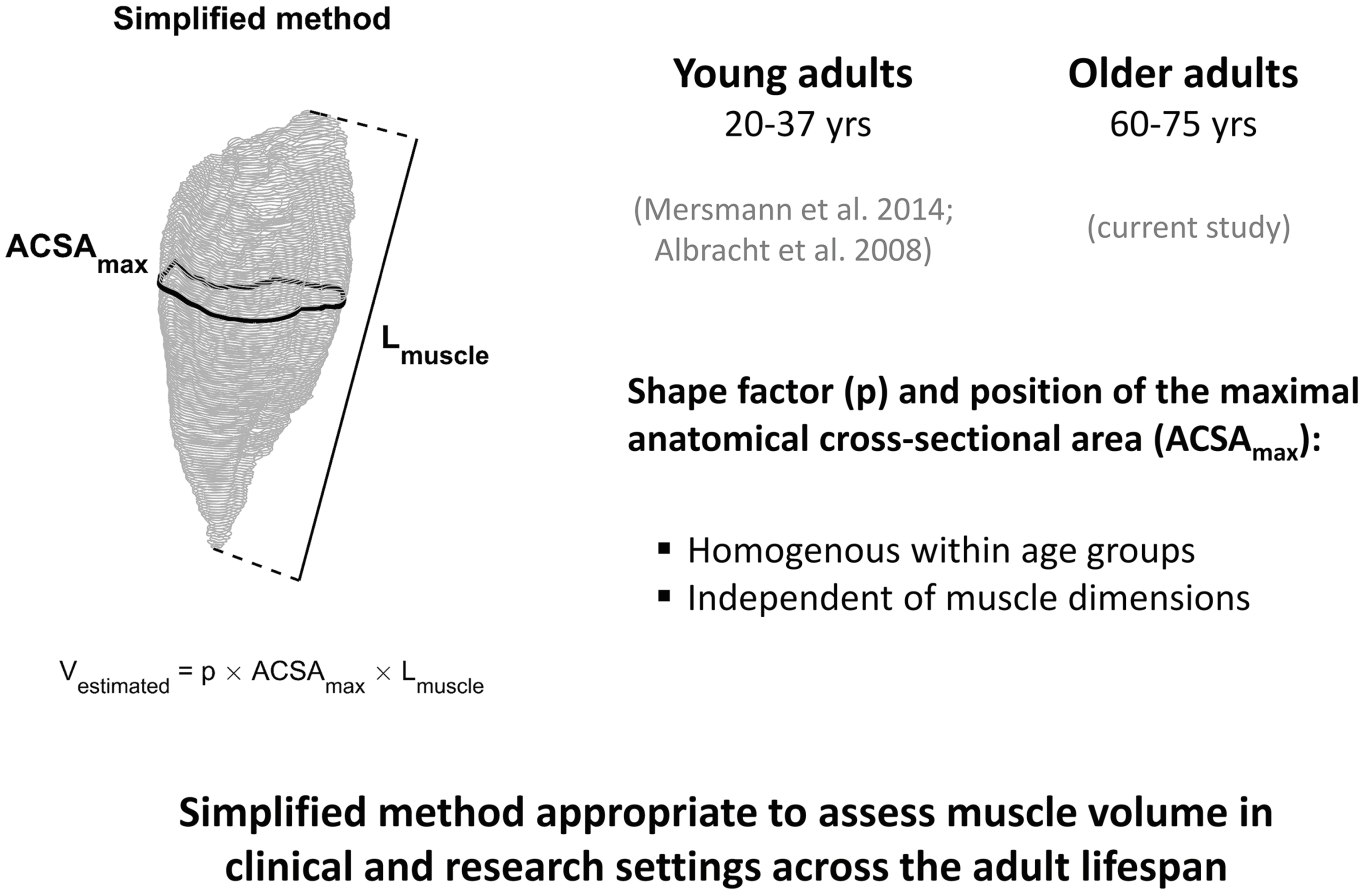
An illustration summarizing the results of the current study in older adults and those from our previous investigations conducted in young adults (Albracht et al., 2008; Mersmann et al., 2014). Based on the observation that the position of the maximum ACSA as well as the muscle specific shape factor does not depend on muscle dimensions and is homogeneous within age groups, we propose that the simplified method is valid for assessment of muscle volume across the adult lifespan. Implementation of this method can greatly reduce the time required for muscle volume assessment compared with full muscle segmentation, an important consideration for clinical and research settings, especially in large cohort studies.

A limitation of the current study may be the relatively low number of participants (*n* = 21), which reduces the potential for detecting statistical differences between methods. Due to the fact that the inter-subject variation in shape factors and the location of the maximum ACSA relative to the shank were very low for the analyzed muscles (coefficient of variation was on average less than 6%), we do not think that increasing the number of subjects would lead to meaningful alterations to our main conclusions. Regarding this, we wish to point out that there was a statistically significant overestimation for GM and SO muscle volumes when using the average shape factor from the young but not from the older adults. Although we found this overestimation of muscle volumes for older adults when using average muscle shape factors from young adults, the RMS differences were rather low. However, it is important to note that the participants in the current study were on average 65 years of age; hence, it seems possible that such differences may be even more pronounced in frail older adults with more advanced age-related atrophy. Combining the current results with those from our previous investigations conducted in young adults (Albracht et al., 2008; Mersmann et al., 2014), we propose that the simplified method is valid for assessment of muscle volume in clinical and research settings across the adult lifespan (Figure 5).

In conclusion, the results of the present study demonstrate that the previously proposed simplified method for assessing TS muscle volume by using the maximal cross-sectional area, muscle length, and a muscle-specific shape factor is applicable for scientific and clinical use in the older population. However, we found evidence for age-specific shape factors and hence limited generalizability among different subject groups, indicating that aging may not only be accompanied by overall muscle atrophy but also a change in the shape of skeletal muscles.

## Data Availability Statement

The data supporting the conclusions of this manuscript will be made available by the authors, without undue reservation, to any qualified researcher.

## Ethics Statement

The studies involving human participants were reviewed and approved by Ethics committee of the German Sport University Cologne. The patients/participants provided their written informed consent to participate in this study.

## Author Contributions

KK and AA contributed to conception of the work. KK and GE performed the data acquisition. KK drafted the manuscript. KK and GE prepared the figures. All authors contributed to analysis and interpretation, approved the final version of the manuscript, and agreed to be accountable for the work.

### Conflict of Interest

The authors declare that the research was conducted in the absence of any commercial or financial relationships that could be construed as a potential conflict of interest.
